# Topic modeling and sentiment analysis of Chinese people’s attitudes toward volunteerism amid the COVID-19 pandemic

**DOI:** 10.3389/fpsyg.2022.1064372

**Published:** 2022-11-04

**Authors:** Ruheng Yin, Jing Wu, Rui Tian, Feng Gan

**Affiliations:** ^1^School of Art, Culture and Tourism Industry Think Tank Chinese Art Evaluation Institute, Southeast University, Nanjing, China; ^2^School of Sociology and Population Studies, Nanjing University of Posts and Telecommunications, Nanjing, China

**Keywords:** volunteerism, COVID-19, China, Weibo, topic modeling analysis, LDA, sentiment analysis

## Abstract

The COVID-19 pandemic has created an urgent need for volunteers to complement overwhelmed public health systems. This study aims to explore Chinese people’s attitudes toward volunteerism amid the COVID-19 pandemic. To this end, we identify the latent topics in volunteerism-related microblogs on Weibo, the Chinese equivalent of Twitter using the topic modeling analysis *via* Latent Dirichlet Allocation (LDA). To further investigate the public sentiment toward the topics generated by LDA, we also conducted sentiment analysis on the sample posts using the open-source natural language processing (NLP) technique from Baidu. Through an in-depth analysis of 91,933 Weibo posts, this study captures 10 topics that are, in turn, distributed into five factors associated with volunteerism in China as motive fulfillment (*n* = 31,661, 34.44%), fear of COVID-19 (*n* = 22,597, 24.58%), individual characteristic (*n* = 17,688, 19.24%), government support (*n* = 15,482, 16.84%), and community effect (*n* = 4,505, 4.90%). The results show that motive fulfillment, government support, and community effect are the factors that could enhance positive attitudes toward volunteerism since the topics related to these factors report high proportions of positive emotion. Fear of COVID-19 and individual characteristic are the factors inducing negative sentiment toward volunteerism as the topics related to these factors show relatively high proportions of negative emotion. The provision of tailored strategies based on the factors could potentially enhance Chinese people’s willingness to participate in volunteer activities during the COVID-19 pandemic.

## Introduction

The outbreak and rapid spread of the COVID-19 pandemic have caused huge economic and public health burdens around the world, posing substantial threats not only to those directly infected but to societies at large (Weible et al., 2020). Governments were forced to implement policies for social distancing and varying degrees of quarantine or lockdown (Chang et al., 2021), which affected even the most developed public health systems (World Health Organization, 2018), thus creating an urgent need for volunteers to augment overwhelmed public health services (Miao et al., 2021). The promotion of volunteerism amid the COVID-19 pandemic is therefore of great urgency and importance, which arouses the interest of many social scientists.

The available studies show inconsistent findings about the general public’s interest in volunteerism in the face of the COVID-19 pandemic. Some studies report a positive relationship between volunteerism and COVID. For example, Beardmore et al. found that many people were motivated to volunteer to support the public health system amid the COVID-19 pandemic (Beardmore et al., 2020). In some cases, as Trautwein et al. demonstrated, the number of volunteers in the COVID-19 pandemic far exceeded the actual demand (Trautwein et al., 2020). Such enthusiasm for volunteerism also occurred in previous crises such as the European 2015 refugee crisis (Simsa et al., 2019). However, other studies demonstrate opposite findings. A good example is that Tran et al. showed that less than 50% of people were willing to volunteer with activities involving COVID-19 patients in Vietnam based on an online cross-sectional survey of 2,032 participants (Tran et al., 2022). In addition, through a web-based, anonymous survey of 518 respondents (68% response rate), Appelbaum et al. found that 42.3% of U.S. medical students intended to discontinue in-person volunteer activities during the COVID-19 pandemic (Appelbaum et al., 2021).

This study aims to add support to the present literature on the exploration of the general public’s willingness to participate in volunteer activities amid the COVID-19 pandemic. To this end, this study examined Chinese people’s discussions about volunteerism on social media using a computerized machine learning model. The objective of this study is not only to analyze what Chinese people say about volunteerism but is also to identify the factors associated with volunteerism. Hopefully, the findings could be translated into appropriate advice to efficiently promote volunteerism amid the COVID-19 pandemic in China.

The rest of the paper is arranged in five sections. In the second section, we provide an overview of several related studies to this paper and raised three research questions. The third section explains the research methodology applied in this study, where the basic steps of data collection and processing are described. Then, in the fourth section, we present the main results of this study, followed by the fifth section, where all of the results are thoroughly discussed. At the end of the paper is the final section, where we present the conclusion, implications, and limitations of this paper.

## Literature review

Since the outbreak of the COVID-19 pandemic, China has mobilized tremendous volunteer resources to complement its public health system. A large number of volunteers took the initiative to participate in the front-line work of epidemic prevention and control (Gao and Zhang, 2021). Researchers have tended to explore the public attitudes toward volunteerism amid the COVID-19 pandemic in China. For example, Zhu et al. surveyed 1,486 civilians in Jiangsu, China to explore social empathy toward COVID-19 volunteer behavior in Chinese society (Zhu et al., 2022). Chen et al. applied a cross-sectional design with data collected from 128 Chinese Older Adults to investigate the patterns of volunteering among older adults in Hong Kong during the COVID-19 pandemic (Chan et al., 2021). However, despite their contributions, previous studies had several methodological limitations as they were almost exclusively conducted using surveys. First, survey is a time-consuming research method. Traditional surveys usually take months to prepare (Shah et al., 2021). Second, survey data are often biased due to increasingly low response rates (usually well below 10%; Munro, 2018). Moreover, survey data come from answers to pre-designed questions, which makes it difficult to detect the dynamic changes in public attitudes toward volunteerism.

This study seeks to address these research limitations by taking advantage of the large-scale and real-time user-generated data on social media platforms (McCormick et al., 2015). Social media has become one of the main venues for information seeking and sharing (Stieglitz et al., 2018). Content produced on social media not only spreads rapidly in cyberspace but also affects people’s perceptions and beliefs in real life (Vosoughi et al., 2018). Therefore, with the analysis of social media discussions, we can find out Chinese people’s unfettered voice on volunteerism. Weibo, the Chinese equivalent of Twitter was chosen as the data source in this study. With 582 million monthly active users and 252 million daily active users by the first quarter of 2022 (Weibo Quarterly Reports, 2022), Weibo is widely considered as China’s leading social media site. The sheer number of users and the relatively free environment makes Weibo a unique platform to investigate issues involving public attitudes in China. For example, Wu et al. extracted Weibo texts to examine the attitudes of the Chinese public toward environmental policies (Wu et al., 2021). Lyu et al. used Weibo data to explore public attitudes of child abuse in mainland China (Lyu et al., 2020). To our knowledge, Weibo has not been tapped as a data source to explore public attitudes toward volunteerism in China.

With the advent of natural language processing (NLP) techniques, researchers have developed a variety of methods to process and analyze Weibo texts, such as Word Cloud, semantic network, and topic modeling. Word Cloud and semantic network process data on the evaluation of word frequency (Gao et al., 2021; Pan et al., 2021). In comparison, topic modeling is a more advanced method as it outputs topics rather than independent words (Blei et al., 2003), allowing researchers to glean more actionable insight from the data (Ramage et al., 2009). We used Latent Dirichlet allocation (LDA) to conduct topic modeling analysis on volunteerism-related Weibo posts in this study. LDA uses a word-based model that treats segmented words as basic units of operations. Each segmented word is associated with a fixed-length vector representation, through which textual information is transformed into numerical content that can be easily modeled by computers. At present, LDA is the most commonly used topic modeling method (He et al., 2020). Researchers have utilized the LDA model to study consumer attitudes (Wang et al., 2022), user feedback (Shi et al., 2022), gender differences (Sun et al., 2020), and many more. To our knowledge, however, this study is the first attempt to use the LDA model to investigate public attitudes toward volunteerism in China.

To further identify the public sentiment toward the latent topics output from volunteerism-related Weibo posts by the LDA model, this study also conducted sentiment analysis on the sample posts. Sentiment analysis is a desirable method for opinion mining and subjectivity analysis (Liu, 2010), which has long been used to help reveal people’s attitudes, opinions, and emotions (Liu and Zhang, 2012). In recent years, there has been a growing prevalence of the utilization of sentiment analysis on social media texts. A good example is that Zheng et al. tracked the evolution of public sentiment in Wuhan, China, during the first 12 weeks of the COVID-19 pandemic using Weibo texts (Zheng et al., 2021). Previous literature also shows that the combination of topic modeling and sentiment analysis is particularly effective in analyzing social media data. For example, Dahal et al. conducted topic modeling and sentiment analysis on global climate change tweets (Dahal et al., 2019). Kwon et al. also combined the two methods to examine online reviews for airlines (Kwon et al., 2021).

This study grows out of the conviction that conducting topic modeling and sentiment analysis on the textual content generated from Weibo offers us a vantage point to contribute to the understanding of Chinese people’s attitudes toward volunteerism amid the COVID-19 pandemic. Specifically, what are the main topics discussed by the Chinese people about volunteerism amid the COVID-19 pandemic, what are the sentiments expressed in the different topics, and what factors associated with volunteerism can be identified based on the topics? The research question is therefore framed as follows:

RQ1. What model of public opinions about volunteerism can be developed?
RQ2. What was the pattern of public sentiments toward volunteerism expressed in the public opinion model?
RQ3. What factors associated with volunteerism can be identified based on the public opinion model?

**Figure 1:**
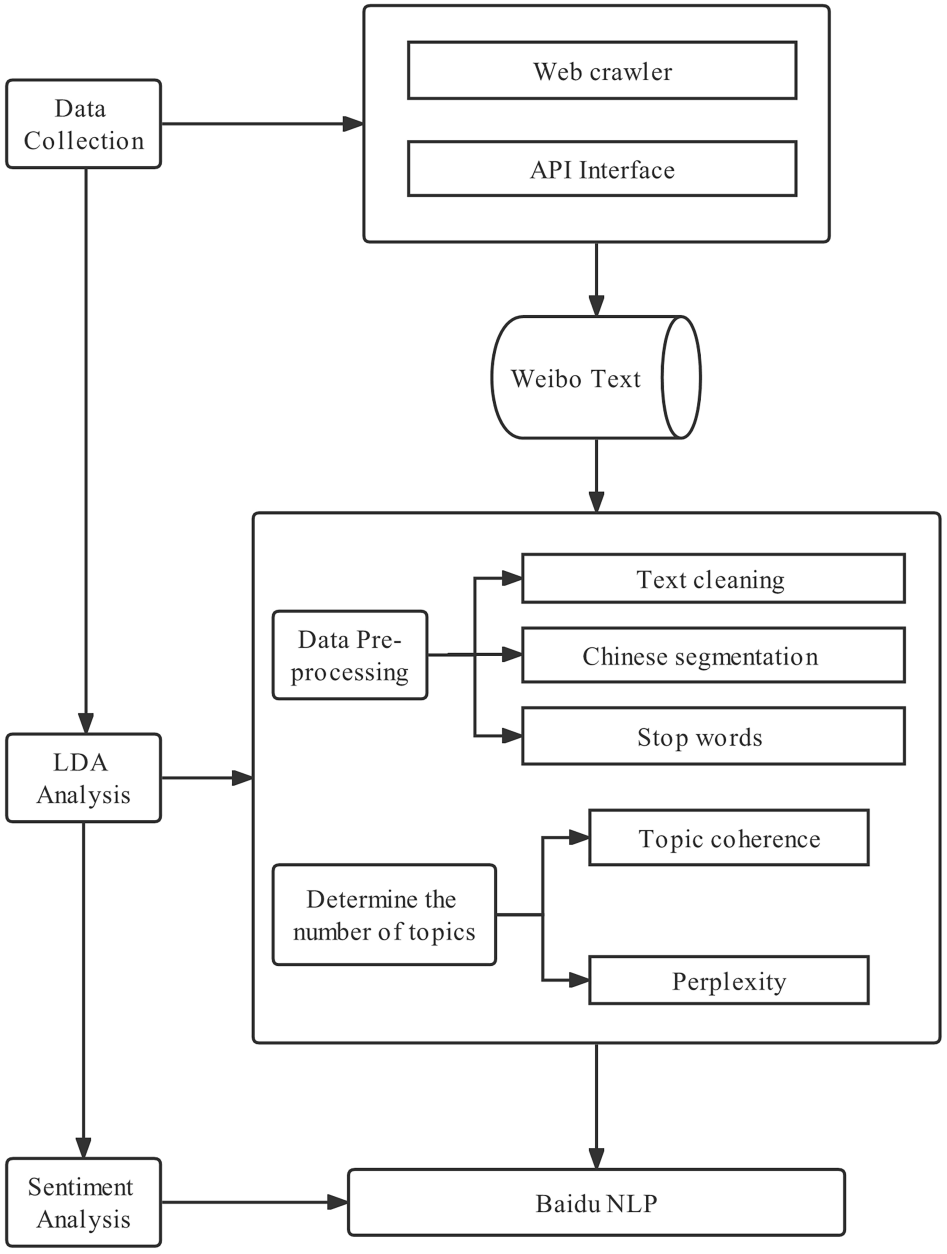
Framework of the data analysis process.

To achieve the research objective, this study proposes an effective research model to mine and analyze data (Figure 1). The findings could serve as a trustworthy reference for policymakers to enhance the willingness of the Chinese people to participate in volunteer activities amid the COVID-19 pandemic.

## Materials and methods

### Data collection and cleaning

Data collection began with defining the research time frame. A fierce COVID-19 outbreak in the city of Shanghai began on March 2022 and ended on August 2022 (China Daily, 2022). Over 25 million people were quarantined at home. The Chinese government mobilized various social forces, volunteers included, to participate in the emergency response system, which sparked heated discussions about volunteerism on Weibo, providing us with enormous data regarding Chinese people’s attitudes toward volunteerism amid the COVID-19 pandemic. Thus, volunteer-related microblogs posted between March 2022 and August 2022 were identified as the sample posts for this study.

The next step was to determine Weibo search terms. We extracted Weibo posts under several different search terms using Weibo public streaming application programming interface (API). After checking the number and scope of the extracted Weibo posts with the help of an expert in the field of volunteerism, Volunteer, Volunteerism, and Volunteer Activities were set to be the search terms in this study. Due to Weibo API’s data-fetching restrictions, the initial return of the data was insufficient for this study. Therefore, we also wrote a web crawler with Python code to circumvent the data-fetching restrictions by simulating logging into Weibo (Yin et al., 2022). In doing so, this study collected 117,867 Weibo posts under the above search terms from March 2022 to August 2022. To ensure the accuracy of the text recognition, we conducted noise reduction in python using Pandas to delete posts that were repeated, meaningless, or consisted of emoji only. In addition, we used prepDocument and plotRemoved to identify and remove low-frequency words. The final dataset included 91,933 Weibo posts.

### Topic modeling analysis

Our primary research objective is to capture the latent topics related to volunteerism on Weibo amid the COVID-19 pandemic. To this end, this study utilized the LDA algorithm to process volunteerism-related Weibo posts. LDA is a three-layer, Bayesian probabilistic generative topic model that is highly effective in identifying latent semantic topics in unstructured textual data, such as Weibo texts (Bicalho et al., 2017).

The first step is to pre-process sample posts in preparation for the text recognition in LDA. First, we used Jieba (a Python package used for Chinese language processing) to conduct word segmentation on the sample posts. Word segmentation refers to splitting a chunk of text into individual words, which is usually the first step to process Chinese texts due to the lack of delimiters between words in written Chinese sentences (Li and Yuan, 1998). Then, we removed common Chinese stop words such as “a,” “the,” or “very,” to generate more meaningful results.

Another critical step to do the LDA model setup is to identify the optimal topic number, which is usually set to achieve the most substantive interpretation of the outcomes rather than the maximization of the topic (Liu et al., 2022). This study used topic coherence and perplexity to measure the optimal number of topics. The topic coherence metric represents the degree of semantic similarity for different topics (Stevens et al., 2012). The perplexity metric measures the performance of a model based on the learned set of topics (Brzustewicz and Singh, 2021). An analysis of topic coherence and perplexity conducted for the number of topics ranging from 0 to 20 revealed 10 as a satisfactory number of topics in this study because this number showed the highest levels of topic coherence and lowest levels of perplexity (Tang et al., 2019).

After data were thoroughly pre-processed and topic number was carefully defined, we embedded the refined dataset into Python using one-hot encoding, which is an effective word embedding tool for topic modeling models such as STM and LDA (Yin et al., 2022). We then ran the topic modeling algorithm using Gensim’s LDA (ŘehůŘek and Sojka, 2010) and obtained the LDA model. The model consists of a mixture of topics and each topic can be represented as a multinomial probability distribution over words (Tang et al., 2019).

### Sentiment analysis

This study is of particular interest to investigate the public sentiment expressed in the topics generated by LDA. For this reason, we conducted sentiment analysis on volunteerism-related Weibo. In particular, we used the open-source natural language processing (NLP) technique from Baidu to automatically label the sentiment polarity of Weibo posts in the final dataset (Cambria et al., 2013). Research has confirmed that this technique is very effective in the sentiment classification of Weibo texts (Tian et al., 2020). Baidu NLP technique treats each Weibo post as a unit of analysis. Each of the sample posts was tagged with a numerical sentiment value from 0 to 1, with 0 indicating a strong negative emotion, and 1 indicating a strong positive emotion. To be specific: values greater than 0.5 indicate positive sentiment, while values less than 0.5 indicate negative sentiment. Values near 0.5 stand for neutral sentiment.

## Results

### General description

**Figure 2:**
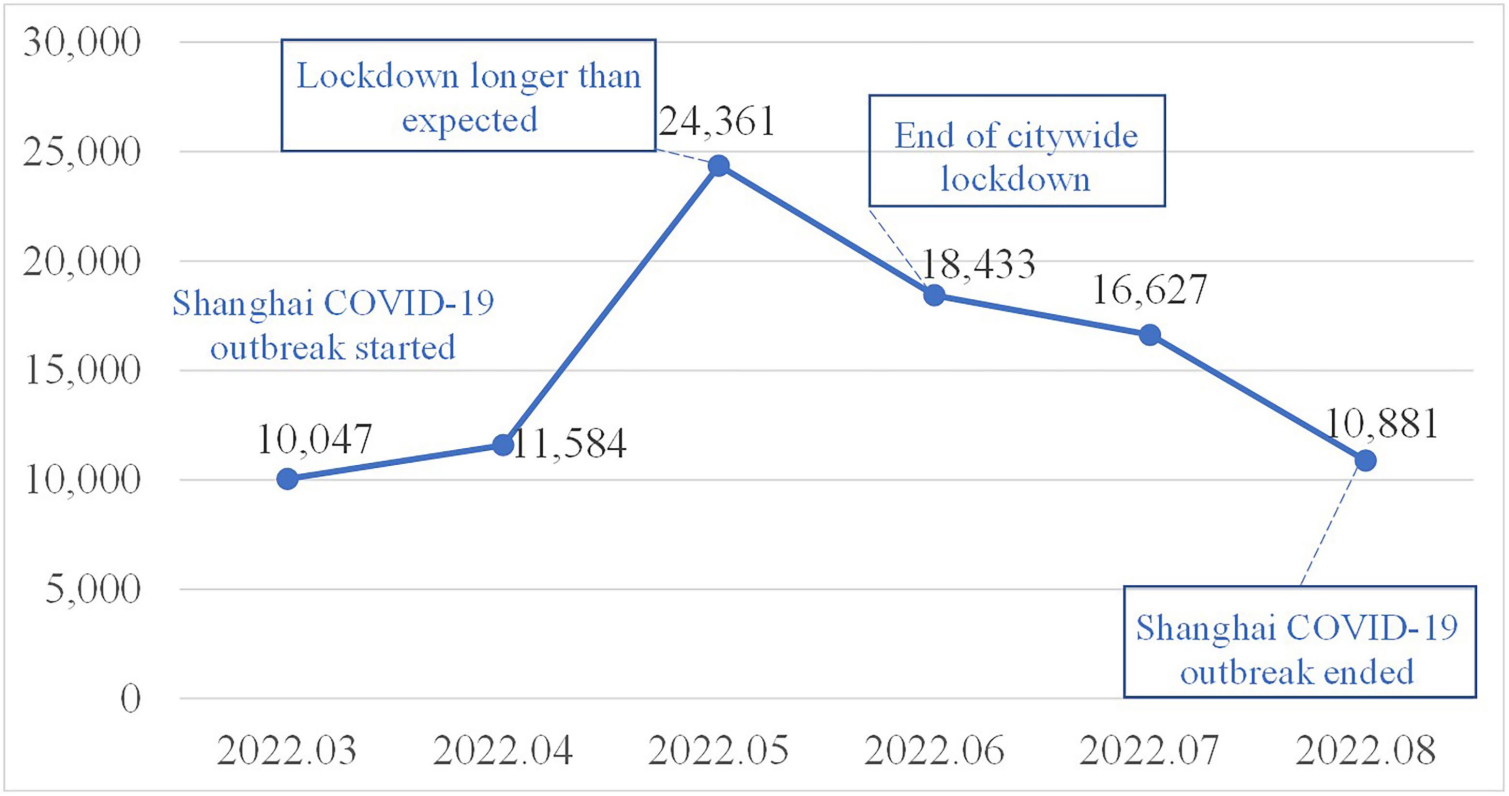
Total number of Weibo posts from March 2022 to August 2022.

Figure 2 shows the total number of Weibo posts under the search terms Volunteer, Volunteerism, and Volunteer Activities during the study period. In general, the number of volunteerism-related Weibo posts fluctuated over the months, with a peak in May. Two months into the complete lockdown in Shanghai, nearly 25,000 volunteerism-related Weibo posts were identified in May 2022, representing a 2-fold increase over the previous month. The lifting of the 75-day Shanghai lockdown on June 1 did not seem to reduce Weibo users’ discussions on volunteerism. Nearly 20,000 volunteerism-related Weibo posts were captured in June. The number of volunteerism-related Weibo posts did not appear to significantly decrease until the end of Shanghai COVID-19 outbreak in August 2022.

### Topic summary

To address the first research question about the model of public opinions, Table 1 shows the 10 topics generated from the LDA model based on the volunteerism-related Weibo posts we extracted from March 2022 to August 2022. The first column shows the topic number. The second column represents the topic label. The labels are not generated automatically in the LDA. Rather, they are assigned by the researchers based on the keywords linked to each of the topics (Sutherland et al., 2020). In this study, a specified label was given for each topic using the authors’ unanimous judgments through an open discussion with one expert in the field of volunteer activities one on one hand, and one expert in the field of emergency management on the other. The third column shows the proportion of each topic. The fourth column demonstrates the keywords. In line with previous literature (Luo et al., 2017), we chose the five highest probability words of each topic as the keywords that, collectively, conveyed the themes of the specific topic.

**Table 1:** Topics of volunteer-related Weibo posts generated by LDA.

**Number**	**Labels**	**Proportion**	**Keywords**
1	Contribution	14.63%	Contribution, help, motive, honor, fulfillment
2	Contagious Nature	14.14%	Infection, contagious, quarantine, lockdown, family
3	Personal Health Conditions	12.51%	Age; conditions; chronic; symptomatic; history
4	Involvement	11.67%	Decision-making; policy; involve; together; transparent
5	Protocol/Guidance	11.28%	Protocol; instruction; rule; guide; help
6	Daily Case Numbers	10.44%	Number; increase; death toll; rapid; update
7	Public Promotion	8.14%	Promotion; pride; celebrity; video; hero
8	Lack of Training/Knowledge	6.73%	Training; knowledge; ignorance; lack; lesson
9	Working Conditions	5.56%	Conditions; food; rest; fatigue; bed
10	Loneliness	4.90%	Communication; friend; community; people; alone

Contribution (topic 1) represents Chinese people’s willingness to contribute to nonprofit works amid the COVID-19 pandemic. The terms include contribution, honor, fulfillment, etc. Contagious nature (topic 2) reflects the worrisome of the Chinese people about potentially being infected at volunteer activities. The terms include infection, contagious, quarantine, etc. Personal health conditions (topic 3) shows that Chinese people with advancing age or chronic conditions could take extra cautious in participating in volunteer activities. The terms include disease, chronic, age, etc. Involvement (topic 4) demonstrates that Chinese people want to be involved in the decision-making process of volunteer activities. The terms include decision-making, policy, involve, etc. Protocol and guidance (topic 5) is about the need for necessary protocol and guidance for volunteer work. The terms include protocol, instruction, guide, etc. Daily case numbers (topic 6) shows that the increased daily updated case numbers could put a damper on Chinese people’s enthusiasm for volunteerism. The terms include number, increase, death toll, etc. Public promotion (topic 7) shows that the promotion of volunteers and volunteer activities on media platforms could encourage Chinese people to participate in volunteerism during the COVID-19 pandemic. The terms include promotion, pride, celebrity, etc. Lack of training and knowledge (topic 8) demonstrates that the lack of basic training and necessary knowledge for volunteer activities could prevent Chinese people from becoming volunteers. The terms include training, knowledge, ignorance, etc. Working conditions (topic 9) is about the importance of decent working conditions for the improvement of the public attitudes toward volunteers. The terms include conditions, food, rest, etc. Loneliness (topic 10) shows that the impact of the COVID-19 pandemic on isolation and loneliness could motivate Chinese people to become volunteers. The terms include communication, community, alone, etc.

To better understand the meaning of the topics, we also examined the most representative Weibo posts of each clustered topic. Table 2 shows the topics and the associated Weibo posts.

**Table 2:** Most representative Weibo texts and topic label selection.

**Contribution (topic 1)** “I really want to help my community through volunteer activities.”; “I want to make actual contributions to prevent and control the virus.”; “Becoming a volunteer has been the most rewarding thing I’ve ever done in my life.”
**Contagious Nature (topic 2)** “Let us be absolutely honest here. No one wants to get infected at volunteer activities.”; “I love to help others. If the virus wasn’t contagious, I would have participated in the volunteer activities.”; “I cannot risk infecting my whole family to participate in volunteer activities.”
**Personal Health Conditions (topic 3)** “At my age, I would almost certainly have been infected if I had been involved in the act of volunteering.”; “I’m too old to participate in volunteerism. They should encourage the young people to do it”; “People who have chronic conditions should not participate in volunteer activities.”
**Involvement (topic 4)** “Volunteers are part of the public health system now.”; “It’s really good to have feedback channels to report the difficulties in the front-line volunteer works.”; “Volunteers are not robots. We need to be involved in the decision-making process.”
**Protocol/Guidance (topic 5)** “The protocols are really helpful.”; “I had no idea what to do when I first became a volunteer. Fortunately, we had clear protocols”; “This is exactly the guidance we need at volunteers activities amid the pandemic.”
**Daily Case Numbers (topic 6)** “I tried not to follow the daily case numbers. This is really discouraging for us volunteers.”; “I want to become a volunteer. But then I look at today’s case numbers.”; “The death toll just keeps counting. I’m terrified to participate in volunteer activities.”
**Public Promotion (topic 7)** “Wow! I cannot believe I’m on TV. This is so cool to speak on behalf of so many volunteers out there!”; “They really make the volunteers look like superheroes.”; “Celebrity effect could motivate more people to become volunteers.”
**Lack of Training/Knowledge (topic 8)** “How can I become a volunteer? I know nothing about volunteerism.”; “I have absolutely no idea of how to do volunteer work. I think I’ll have to pass.”; “They should only use experienced volunteers during the pandemic.”
**Working Conditions (topic 9)** “The food here is amazing. Kudos to whoever in charge of this.”; “There’s nothing to complain about the work conditions. They did what they can do.”; “We can even take a nap from time to time. This is great!”
**Loneliness (topic 10)** “It feels so good to talk to people. I guess that’s the perk in volunteerism.”; “I’m so sick of the quarantined life. I cannot stand the loneliness. That’s why I become a volunteer.”; “I met some friends at volunteer activities. This really is the best way to overcome loneliness.”

### Sentiment analysis

To investigate the public sentiment toward the 10 topics extracted from Weibo discussions on volunteerism, this study also conducted sentiment analysis on the sample posts. Sentiment analysis has three sentiment classifications: dichotomous (positive and negative), trichotomous (positive, neutral, negative), and quadratic (positive, neutral, negative, and other; Salathé and Khandelwal, 2011). During the labeling process, we found that most of the Weibo posts were classified into positive and negative categories, and there were very few Weibo posts identified as neutral (less than 4%). Thus, this study chose dichotomous [positive (non-negative, including neutral) and negative].

**Figure 3:**
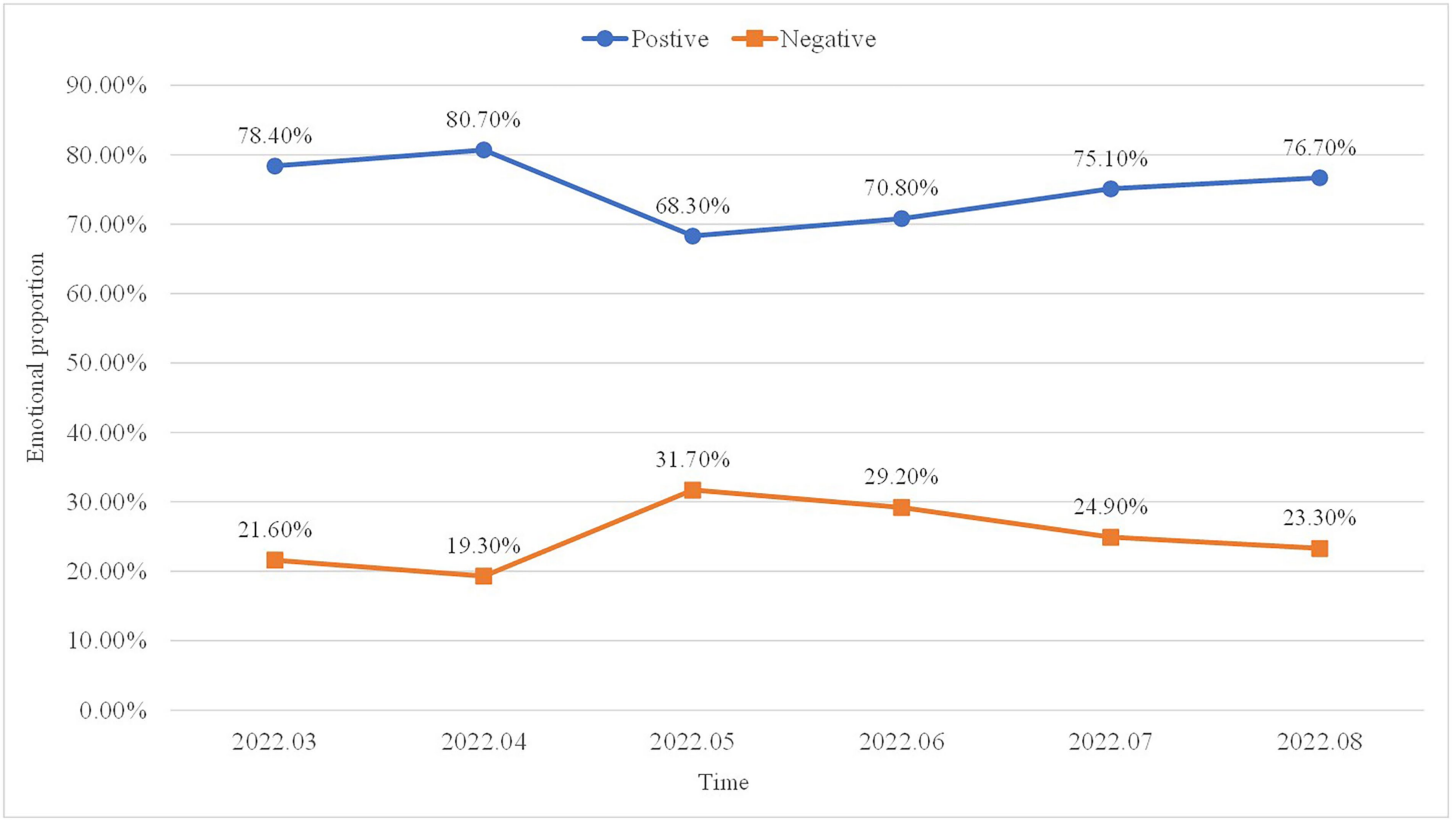
Variations in sentiment values from March 2022 to August 2022.

With regard to the second research question on the pattern of public sentiments toward volunteerism in China, Figure 3 shows the variations in sentiment values on volunteerism from March 2022 to August 2022. Overall, Chinese people held an open attitude toward volunteerism amid the COVID-19 pandemic. Over 75% of the Weibo posts were labeled as positive throughout the study period, and over 70% were positive in most of the study period. An observable degree of sentiment fluctuation was illustrated from the shift in the percentages of positive and negative Weibo posts over the four-month study period. In certain months (i.e., March 2022 and April 2022), the Chinese public perceived volunteerism significantly positive, yet in other months (i.e., May 2022 and June 2022), public perceptions were rather negative compared to the average sentiment.

**Figure 4:**
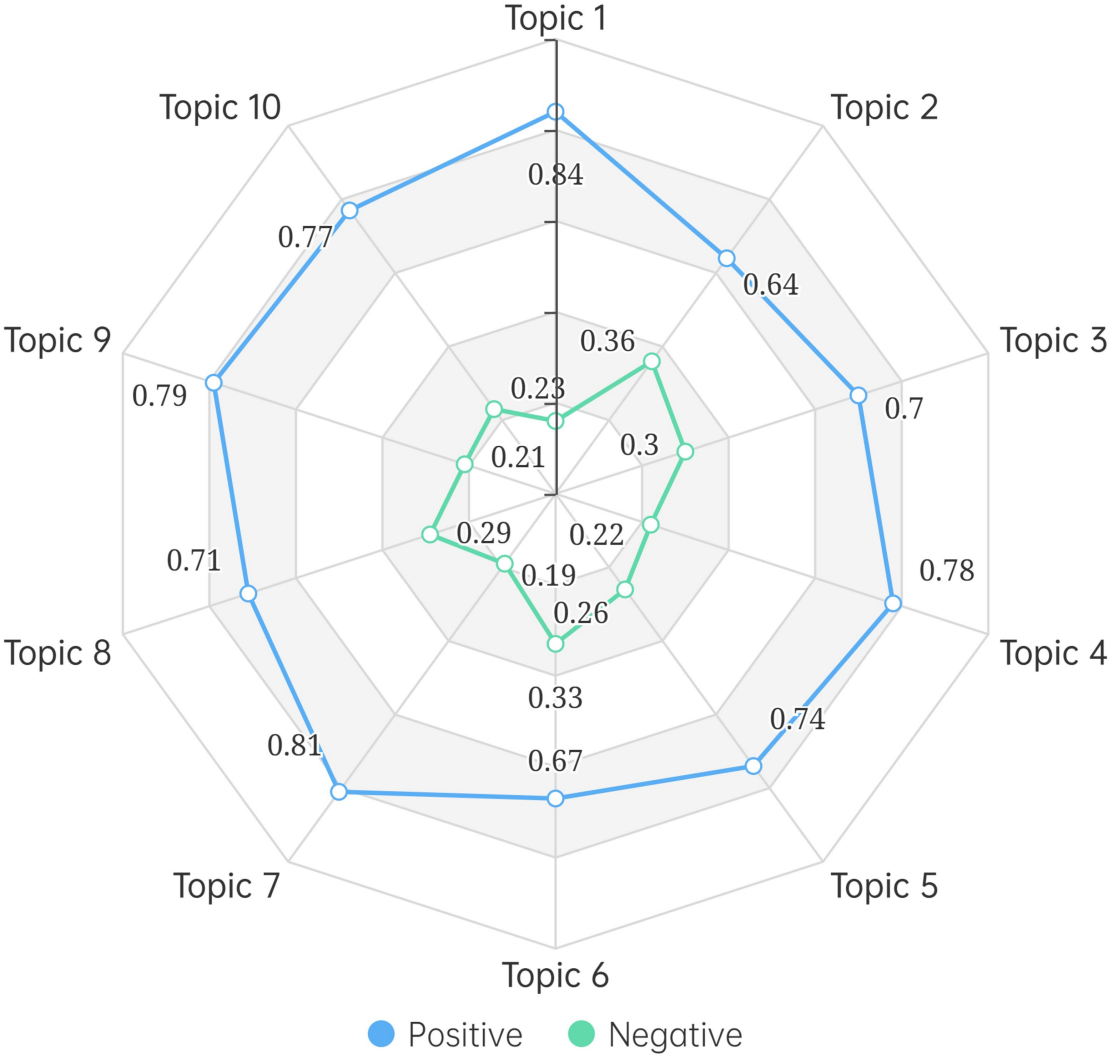
Topic–sentiment distribution of Weibo posts.

Sentiment data were reorganized to demonstrate the public sentiment toward the 10 topics extracted from sample posts. The topic–sentiment distribution is shown in Figure 4, which illustrates the percentages of positive and negative emotions across the 10 topics. Overall, the 10 topics demonstrate a diverse sentiment distribution. It also comes to the attention of this study that positive emotion accounts for the greatest proportion of Weibo posts on each topic. In particular, contribution (topic 1) and public promotion (topic 7) report higher percentages of positive emotions, which are 84 and 81%, respectively. This finding indicates that discussions around these two topics are most likely to motivate Chinese people to engage in volunteer activities. In comparison, contagious nature (topic 2) and daily case numbers (topic 6) record higher proportions of negative emotion, which are 64 and 67% respectively, showing that these two topics are the primary reasons that reduce Chinese people’s willingness to become volunteers during the COVID-19 pandemic.

## Discussion

**Figure 5:**
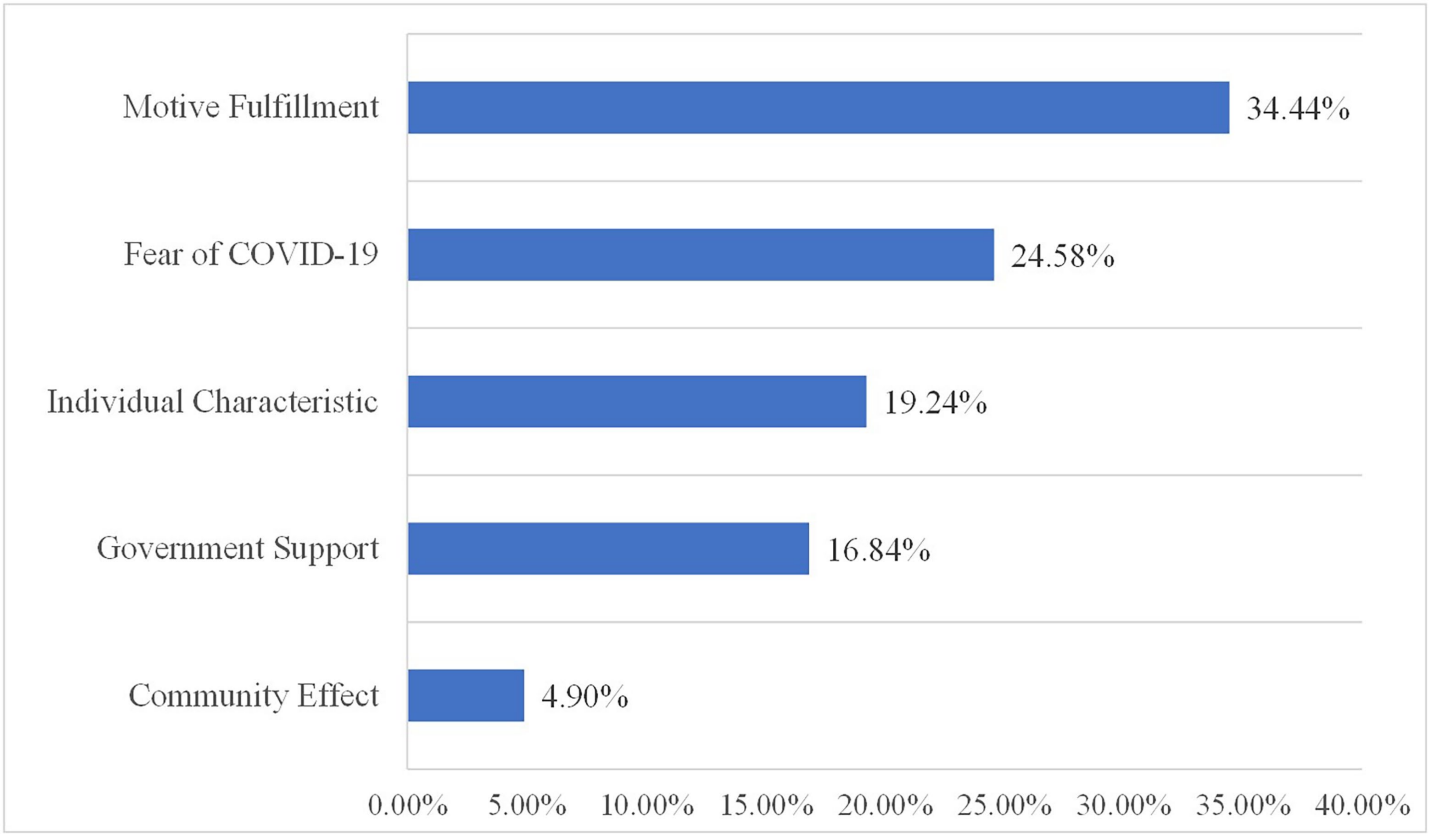
Factors associated with volunteerism amid the COVID-19 pandemic.

Further to explore the third research question about the factors associated with volunteerism amid the COVID-19 pandemic in China, as shown in Figure 5, this study distributes the 10 topics into five factors based on the themes of the topics: motive fulfillment (*n* = 31,661, 34.44%), fear of COVID-19 (*n* = 22,597, 24.58%), individual characteristic (*n* = 17,688, 19.24%), government support (*n* = 15,482, 16.84%), and community effect (*n* = 4,505, 4.90%). In particular, motive fulfillment contains contribution (topic 1), involvement (topic 4), and public promotion (topic 7). Fear of COVID-19 contains contagious nature (topic 2) and daily case numbers (topic 6). Individual characteristic includes personal health conditions (topic 3) and lack of training and knowledge (topic 8). Government support includes protocol and guidance (topic 5) and working conditions (topic 9). Community effect includes loneliness (topic 10).

Motive fulfillment is the primary factor for Chinese people to participate in volunteer activities amid the COVID-19 pandemic. The topics distributed in this category not only account for nearly 35% of Weibo discussions on volunteerism but also represent strong positive sentiment based on the sentiment values. This is in line with previous findings that the main reason that explained general satisfaction with volunteerism was the intrinsic and extrinsic rewards of volunteering (Kulik et al., 2016). Government support is another critical factor that could positively affect COVID-19 volunteerism participation. Almost 17% of the sample posts were clustered into topics related to this factor. The overall proportions of positive emotion are over 80%. This study also found that the community effect arising from volunteer activities could help cope with the feelings of loneliness caused by the COVID-19 pandemic. 77% of the Weibo posts distributed in this factor were identified as positive.

Fear of COVID-19 is the main reason for negative attitudes toward volunteerism amid the COVID-19 pandemic among Chinese people. Nearly 25% of the Weibo discussions fall into this category. The overall proportions of negative emotion are nearly 35%. The negative sentiment expressed in this factor focuses on two aspects. First, the contagious nature of COVID induces anxiety and depression among Chinese people (Ren et al., 2020), which discourages them to participate in volunteer activities. Second, the increased daily case numbers wreak psychological havoc on Chinese people (Liu et al., 2021), which negatively affect Chinese people’s attitudes toward volunteerism. Individual characteristic also demonstrates high proportions of negative emotion. Over 25% of the Weibo posts distributed in this factor were identified as negative. The susceptibility to symptomatic COVID-19 is related to chronic conditions and advancing age (Tisminetzky et al., 2022). Therefore, Chinese people in this demographic could be particularly wary of participating in volunteer activities amid the COVID-19 pandemic. In addition, the lack of necessary training and knowledge also contributes to the relatively negative nature of this factor.

This study proposes the following suggestions to improve public attitudes toward volunteerism amid the COVID-19 pandemic in China. First, policymakers could invite celebrities to publicly promote volunteerism as well as disseminate knowledge of volunteerism. In doing so, the general public could be temporally motivated to participate in volunteerism. A good example is that when Angelina Jolie detailed her decision to undergo a double mastectomy to prevent herself from breast cancer in New York Times column, daily test rates for the breast cancer gene (BRCA) increased by 64% in the next 15 business days (Desai and Jena, 2016). Second, for those who are concerned about getting infected at volunteer activities, policymakers could encourage them to volunteer in non-patient contact activities such as data entry and logistics. These types of volunteer activities are also of great value for the prevention and control of the COVID-19 pandemic.

## Conclusion

Governments throughout the world have put significant efforts into motivating people to volunteer in public health services amid the COVID-19 pandemic. This study contributes to research on the exploration of the public attitudes toward volunteerism and identify several factors that could potentially influence Chinese people’s willingness to participate in volunteer activities amid the COVID-19 pandemic. This study collected data from China’s leading social media site Weibo. After cleaning and pre-processing, the data were put into the LDA to model the latent topics that could reflect the interests and concerns toward volunteerism among Chinese people. In addition, this study conducted sentiment analysis using the open-source NLP technique from Baidu to examine the public sentiment toward the topics. With an in-depth analysis of 91,933 Weibo posts, this study captures 10 topics that are, in turn, classified into five factors, namely, motive fulfillment (*n* = 31,661, 34.44%), fear of COVID-19 (*n* = 22,597, 24.58%), individual characteristic (*n* = 17,688, 19.24%), government support (*n* = 15,482, 16.84%), and community effect (*n* = 4,505, 4.90%). The results show that motive fulfillment, government support, and community effect are the factors that could enhance positive attitudes toward volunteerism amid the COVID-19 pandemic since topics related to these factors demonstrate high proportions of positive emotion. Fear of COVID-19 and individual characteristic are the primary factors inducing negative sentiment toward volunteerism during the COVID-19 pandemic as topics related to these factors show high proportions of negative emotion. The findings provide actionable insights for policymakers to improve the public attitudes toward volunteerism during the COVID-19 pandemic in China.

This study has several positive implications for research in the field of volunteerism. First, compared to the traditional research methods in this field (i.e., questionnaire survey), this study utilizes natural language processing techniques to tap the real-time user-generated data on social media. In doing so, this study garnered an enormous amount of data that is unprecedented in this research field. Second, the adoption of machine learning models to capture and analyze the emotional tendencies toward volunteerism expressed in the Weibo posts helps deepen the understanding of the data, which could unlock information supporting new insights in this research field. Further, although the findings of this study are unique in the Chinese context, they have the potential to be generalized and thus contribute to the research field as a whole.

Every empirical study has limitations. First, Weibo is not the only platform through which Chinese people share their views. Many individuals may express their attitudes toward volunteerism within other platforms. Second, based on the level of volunteers, volunteerism can be divided into three classifications as community-based volunteering, professional volunteering, and emergency volunteering (Gouda et al., 2020). This study did not consider different volunteering groups’ attitudes toward volunteerism during the COVID-19 pandemic, which could be explored in future studies. Third, because Weibo data did not contain information revealing the age of the posters, this study did not analyze different age group’s attitudes toward volunteerism. Future studies could extract data from other social media platforms that contain such information. Last, this study focused solely on textual content. Future studies could also take advantage of the graphic and video content on Weibo.

## Data availability statement

The raw data supporting the conclusions of this article will be made available by the authors, without undue reservation.

## Author contributions

All authors listed have made a substantial, direct, and intellectual contribution to the work and approved it for publication.

## Funding

This research was supported by the Major Project of the National Social Science Fund (21ZD11), Nanjing University of Posts and Telecommunications (NYY220025), and Provincial Social Science Foundation of Hebei (HB22GL022).

## Conflict of interest

The authors declare that the research was conducted in the absence of any commercial or financial relationships that could be construed as a potential conflict of interest.

## Publisher’s note

All claims expressed in this article are solely those of the authors and do not necessarily represent those of their affiliated organizations, or those of the publisher, the editors and the reviewers. Any product that may be evaluated in this article, or claim that may be made by its manufacturer, is not guaranteed or endorsed by the publisher.
